# Genotype II Live-Attenuated ASFV Vaccine Strains Unable to Completely Protect Pigs against the Emerging Recombinant ASFV Genotype I/II Strain in Vietnam

**DOI:** 10.3390/vaccines12101114

**Published:** 2024-09-28

**Authors:** Nguyen Van Diep, Nguyen Van Duc, Nguyen Thi Ngoc, Vu Xuan Dang, Tran Ngoc Tiep, Viet Dung Nguyen, Thi Tam Than, Dustin Maydaniuk, Kalhari Goonewardene, Aruna Ambagala, Van Phan Le

**Affiliations:** 1 AVAC Vietnam Joint Stock Company, Ngoc Lich Village, Trung Trac Commune, Van Lam District, Hung Yen 160000, Vietnam; diep.ngv@gmail.com (N.V.D.); ducnv@avac.com.vn (N.V.D.); ngocnt@avac.com.vn (N.T.N.); vxdang.vet@gmail.com (V.X.D.); tieptn@avac.com.vn (T.N.T.); 2 Faculty of Animal Science and Veterinary Medicine, Bac Giang Agriculture and Forestry University, Bac Giang 230000, Vietnam; nguyendungdhnl@gmail.com; 3 College of Veterinary Medicine, Vietnam National University of Agriculture, Hanoi 100000, Vietnam; thantam207@gmail.com; 4 Canadian Food Inspection Agency, National Centre for Foreign Animal Disease, Winnipeg, MB R3E 3R2, Canada; dustin.maydaniuk@inspection.gc.ca (D.M.); kalhari.goonewardene@inspection.gc.ca (K.G.); 5 Laboratory of Viral Infectious Diseases, Center for Research Excellence and Innovation, Vietnam National University of Agriculture, Hanoi 100000, Vietnam

**Keywords:** ASF, live-attenuated, vaccine, emerging, rASFV I/II, ASFV-G-ΔMGF, ASFV-G-ΔI177L

## Abstract

**Background**: African swine fever virus (ASFV) continues to spread globally, causing severe economic losses to pig farmers. Vietnam licensed two live attenuated vaccines based on the ASFV strains ASFV-G-ΔI177L and ASFV-G-ΔMGF to control the ongoing ASF outbreaks. In 2023, newly emerging highly virulent recombinant ASF viruses (rASFV I/II) containing genetic elements from both p72 genotype I and II ASF viruses were reported from Northern Vietnam. **Objective**: This study evaluated whether the two vaccine strains were able to protect the pigs against the emerging rASFV I/II strain VNUA/rASFV/TN1/23. **Results**: Pigs vaccinated with ASFV-G-ΔMGF or ASFV-G-ΔI177L, when challenged with rASFV I/II, succumbed to the infection, or developed signs of chronic ASF. **Conclusions**: The findings from this study show that both vaccine strains that are licensed and used in Vietnam are unlikely to protect pigs from the emerging highly virulent rASFV I/II. This complicates the ongoing efforts to control ASF in Asia and globally and emphasizes the urgent need for a novel vaccine that can effectively protect pigs from the rASFV I/II.

## 1. Introduction

African swine fever (ASF) is a contagious fatal hemorrhagic disease of domestic and European wild pigs [1]. It continues to spread globally, with serious implications for swine health, trade, and economy. The causative agent, ASF virus (ASFV), is a large DNA virus with complex structure and pathogenicity [2,3]. Historically, based on the partial sequence of the gene B646L encoding ASFV p72 protein, ASF viruses have been grouped into 24 genotypes [4].

Until 1957, ASF was restricted to Sub-Saharan Africa. In 1957, a p72 genotype I ASFV from Angola spread to Portugal [5]. The disease was considered eradicated from Portugal the following year; however, two years later, ASF again appeared in Portugal and subsequently spread to the neighboring countries in Europe [5,6]. Later, the virus spread to Malta, Cuba, Brazil, Haiti, and the Dominican Republic [6]. The disease was successfully eradicated from Europe and the Americas by 1995, except from the Island of Sardinia [6,7]. In June 2007, an ASF outbreak caused by a p72 genotype II ASFV was confirmed in the Caucasus region of Georgia [8]. The virus quickly spread to Armenia (August 2007) and to the Russian Federation (November 2007) and slowly but steadily spread to the neighboring countries [9]. In 2018, the virus entered China, the country with the largest pig population in the world [10]. From China, ASFV p72 genotype II rapidly spread to Vietnam and the neighboring countries in Asia, killing millions of pigs [11].

In 2021, China reported detection of low-virulent p72 genotype I ASFV circulating in domestic pigs [12]. These viruses showed high genetic similarity to the non-hemadsorbing ASF viruses isolated in Portugal in 1968 and 1988. Despite low virulence, they spread efficiently, causing mild onset and chronic disease characterized by necrotic skin lesions and joint swellings. In 2023, highly virulent ASFV strains containing genetic elements of both genotypes I and II ASF viruses emerged in three provinces of China [13]. The recombinant ASFV (rASFV I/II) strains contained ~44% of the genome, including the p72 gene, from the low-virulent genotype I strains and the remaining 56% of the genome from genotype II strains previously reported from China. The live-attenuated p72 genotype II vaccine strain HLJ/18-7GD was not able to protect pigs against the new rASFV I/II [13].

Vietnam reported its first ASFV outbreak in February 2019 in the Northern Hung Yen province [14]. The virus was similar to the p72 genotype II ASF viruses circulating in China, and it spread throughout the entire country within 5 months [15]. Since then, p72 genotype II ASF viruses have been circulating in Vietnam, killing millions of pigs and causing severe economic losses to farmers [16]. In September 2023, rASFV I/II was detected in family-run backyard farms in Northern Vietnam [17]. The rASFV I/II from Vietnam was genetically similar to those reported from China, indicating a recent spread of these viruses from China to Vietnam [17,18].

In 2023, Vietnam licensed two live-attenuated ASFV vaccines, AVAC ASF LIVE^®^ (AVAC Viet Nam JSC, Hung Yen, Vietnam) and NAVET-ASFVAC^®^ (Navetco Central Veterinary Medicine Company, Hanoi, Vietnam), to control the ongoing ASFV outbreaks. The master seed in the AVAC ASF LIVE^®^ was produced by multiple passaging and cloning of ASFV-G-ΔMGF strain [19] in a cell line (DMAC) developed by the AVAC Viet Nam JSC [20]. The NAVET-ASFVAC^®^ vaccine contains the ASFV-G-ΔI177L strain [21] propagated in porcine peripheral leukocyte cultures.

Both these vaccines are recommended for fattening pigs 4 weeks and older, and a single dose induces protection against the ASF p72 genotype II viruses circulating in Vietnam [20,22]. This study was conducted to evaluate the protective efficacy of the two vaccine strains, ASFV-G-ΔI177L and ASFV-G-ΔMGF, against the emerging rASFV I/II strain in Vietnam.

## 2. Materials and Methods

### 2.1. Viruses

The ASFV-G-ΔMGF strain propagated and titrated on the DMAC (Diep’s Macrophage cell) cell line was provided by AVAC JSC. The ASFV-G-ΔI177L strain [20], propagated and titrated on porcine primary leukocyte (PPL) cultures, was obtained from the USDA. The rASFV I/II strain VNUA/rASFV/TN1/23 [18] was propagated and titrated on PPL cultures.

### 2.2. Animal Experiment

A total of 18 healthy, 8-week-old, crossbred (Yorkshire-Landrace-Duroc) pigs obtained from a high-health commercial pig farm in Bac Giang, Vietnam, were used in this study. All pigs were confirmed negative for ASF, porcine circovirus-2, foot-and-mouth disease, classical swine fever, and porcine reproductive and respiratory syndrome viruses by real-time PCR and real-time RT-PCR. Pigs were also tested negative for anti-ASFV antibodies by enzyme-linked immunosorbent assay (VDPro^®^ ASFV Ab i-ELISA Version 2.0 Kit, Median Diagnostics, Seoul, Republic of Korea). Upon arrival, pigs were randomly assigned into three groups (six pigs per group), ear-tagged, and moved into three separate pens at the animal facility at AVAC JSC. Pigs were provided ad libitum feed and water and observed at least once daily before vaccination and twice a day after.

After one week of acclimatization, pigs in the first group (pigs #5–10) were inoculated with 1 mL sterile PBS per pig IM. The second group (pigs #11–16) was vaccinated intramuscularly (IM) with ASFV-G-ΔI177L (10^4^ HAD_50_ in 1 mL per pig). The third group (pigs #17, 18, 20, 21, 22, and 23) was vaccinated IM with ASFV-G-ΔMGF strain (10^4^ HAD_50_ IM in 1 mL per pig). Please note that there was no pig #19. After vaccination, pigs were monitored twice daily for clinical signs, and their rectal temperatures were measured in the morning using a digital thermometer while the pigs were eating. Nasal swabs, serum, and whole blood samples from each pig were collected at 0, 7, 14, 21, and 28 days post-vaccination (dpv). For sample collection, each pig was restrained using a snare. After the sample collection on 28 dpv, all pigs were challenged IM with rASFV I/II at 10^3^ HAD_50_ in 1 mL per pig.

After the challenge, pigs continued to be monitored twice daily for clinical signs (fever, anorexia, depression, skin discoloration, staggering gait, cough, constipation, and diarrhea), and rectal temperatures were measured daily throughout the experiment. Nasal swabs, serum, and whole blood samples were collected at 4, 7, 14, 21, and 28 days post-challenge (dpc). Rectal temperatures above 40 degrees for 2 consecutive days were considered fever. Any pig reaching the humane endpoint was euthanized using 10 mL of sodium pentobarbital (240 mg/mL) intravenous injection. Any animal found dead or euthanized was subjected to a full postmortem examination, and tissues were collected for virus detection. A clinical scoring system developed in-house (Supplementary Materials Table S1) was used to record the daily clinical findings for each animal.

### 2.3. DNA Extraction and Real-Time PCR (RT-PCR)

Total DNA from whole blood, nasal swabs, and tissue samples were extracted using Dneasy Blood & Tissue Kit (Qiagen, Hilden, Germany) following the manufacturer’s instructions. ASFV genomic material was detected using the VDx ASFV qPCR kit (Median Diagnostics) according to the instructions in a CFX96 Touch Real-Time PCR Detection System (Bio-Rad Laboratories Ltd., Hercules, CA, USA).

To differentiate the vaccine strains from the rASFV I/II strain, two RT-PCR assays, Phan G1 and Phan G2, were developed. Both assays target the ASFV p72 gene and use the same forward and reverse primers but two different probes (Supplementary Materials Table S2A). The Phan G1 probe is specific for the ASFV p72 gene encoded by genotype I viruses and the rASFV I/II strain. The Phan G2 probe is specific for p72 genotype II viruses. The reaction conditions were 50 °C for 5 min, 95 °C for 20 s, followed by 40 cycles of 95 °C for 3 s and 60 °C for 30 s. Samples with a Ct (cycle threshold) value less than 40 were considered positive for ASFV genomic material. The sensitivity of the Phan G1 and G2 assays were compared to the pan-ASFV real-time PCR assay Tignon [23] and ASFV Genotype I specific real-time PCR assay G1-ACDP [24] (Supplementary Materials Table S2B).

### 2.4. ELISA

Anti-ASFV antibodies in the serum samples were detected using VDPro^®^ ASFV Ab i-ELISA V 2.0 kit (Median Diagnostics) according to the instructions from the manufacturer. Samples with result interpretation S/P ratios ≥ 0.25 were considered positive, while those with S/P ratios < 0.25 were considered negative.

## 3. Results

### 3.1. Clinical Signs

**Figure 1:**
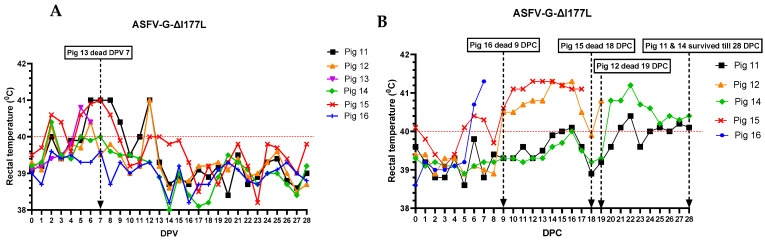
Daily rectal temperatures of pigs (#11–16) inoculated with ASFV-G-ΔI177L (10^4^ HAD_50_ IM per pig) before (**A**) and after (**B**) challenge with rASFV I/II (10^3^ HAD_50_ IM per pig). The dates pigs were found dead or euthanized as they reached the humane endpoint are marked with an arrow. DPV = days post-vaccination. DPC = days post-challenge.

Of the six pigs vaccinated with ASFV-G-ΔI177L, three (pigs #11, 13, and 15) developed a mild fever between 2–5 dpv (Figure 1A), and pigs #16 and #11 were lethargic and not interested in feed between 5–7 dpv.

**Figure 2:**
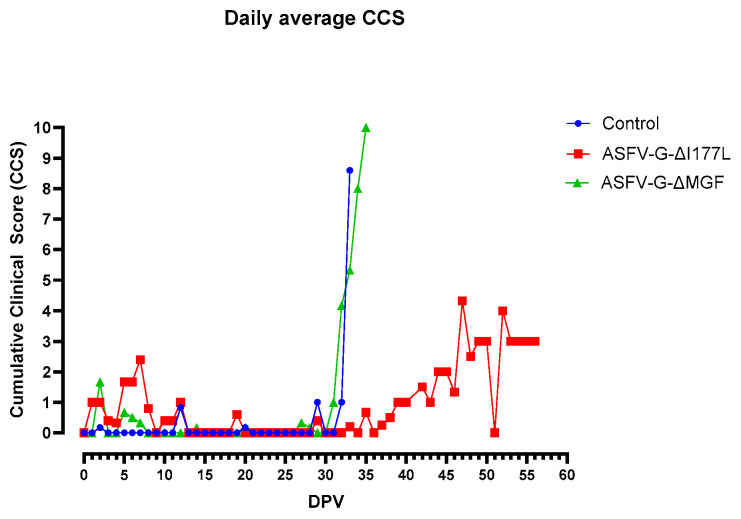
Daily average cumulative clinical scores (CCS) of the pigs that received ASFV-G-∆I177L, ASFV-G-ΔMGF, or PBS before and after the challenge with the rASFV I/II strain. DPV = days post-vaccination.

As a result, the daily cumulative clinical score (CCS) of this group increased slightly (Figure 2).

**Figure 3:**
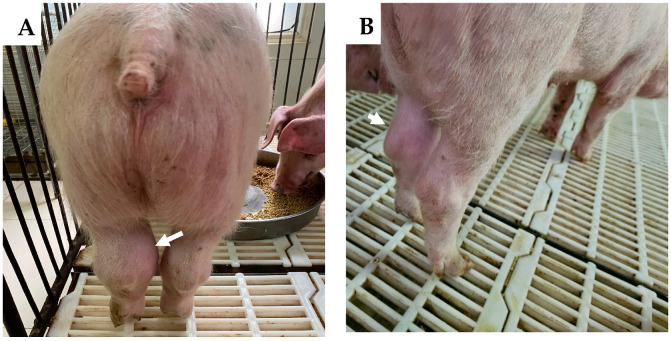
Swelling of tarsal joints (arrows) of pigs #14 (**A**) and #11 (**B**) that survived the rASFV I/II challenge.

At 7 dpv, pig #13 was found dead. After challenge with the rASFVI/II, pig #15 developed a mild fever at 5 dpc, but the rectal temperature returned to normal by 8 dpc. Starting at 9 dpc, the rectal temperature of pig #15 increased again, reached its peak by 12 dpc, and remained high until it was found dead at 18 dpc. Pig #16 developed fever at 6 dpc, reached 41.5 °C by 7 dpc, and was found dead at 9 dpc. Pig #12 developed fever at 9 dpc, peaked at 14 dpc, returned to normal at 18 dpc, developed fever again at 19 dpc, and was euthanized on the same day as it reached the humane endpoint. Pigs #14 and 11 developed a mild fever at 20 and 21 dpc, respectively, and the fever remained until the end of the study. As the pigs developed clinical signs post-challenge, the CCS of the group increased gradually (Figure 2). Starting at 20 dpc, pig #14 started limping, and a swelling in its left hock (tarsal) joint was observed. It became lethargic and depressed, and the swelling and limping worsened over time. By 28 dpc, a mild swelling in the right hock joint and a large subcutaneous hemorrhage with denuded skin were observed under the neck. Pig #11 stayed active until the end of the study but started to develop a mild swelling in the left hock joint around 27 dpc (Figure 3). Pigs #14 and 11 were euthanized at 28 dpc in order to conclude the study.

**Figure 4:**
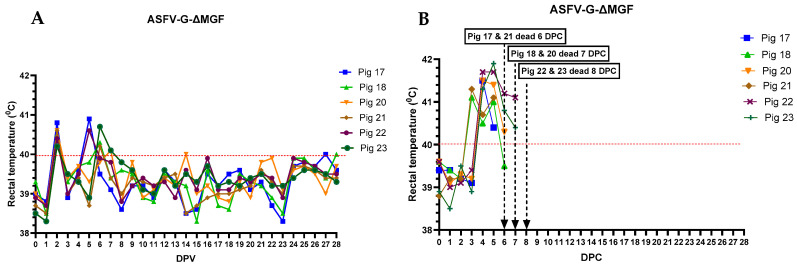
Daily rectal temperatures of pigs (#17–23) inoculated with ASFV-G-ΔMGF (10^4^ HAD_50_ IM per pig) before (**A**) and after (**B**) the challenge with rASFV I/II (10^3^ HAD_50_ IM per pig). The dates pigs were euthanized are marked with an arrow. DPV = days post-vaccination. DPC = days post-challenge.

In the ASFV-G-ΔMGF group, one pig (#23) developed a mild fever for 2 days (Figure 4A). All pigs remained active and alert until they were challenged at 28 dpv; hence, the CCS of these animals remained low (Figure 2). After the challenge, all six pigs developed high fever, anorexia, lethargy, and skin hemorrhages and were humanely euthanized by 8 dpc (Figure 4B).

**Figure 5:**
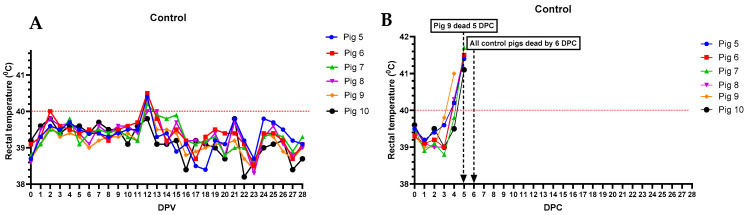
Daily rectal temperatures of control pigs (#5–10) inoculated IM with a sterile PBS (**A**) and challenged with rASFV I/II (10^3^ HAD_50_ IM per pig) (**B**). The dates pigs were found dead or euthanized are marked with an arrow. DPV = days post-vaccination. DPC = days post-challenge.

As expected, all pigs in the control group (pigs #5–10) remained bright and alert, exhibited normal appetite, and developed no fever before the challenge (Figure 5A). After the challenge, all six pigs developed high fever (Figure 5B), severe anorexia, and lethargy and were found dead or humanely euthanized within 6 days. No skin lesions, respiratory (labored breathing), or digestive (constipation, diarrhea, or blood in stools) signs were observed before they succumbed to ASF.

### 3.2. Viremia and Virus Shedding

**Figure 6:**
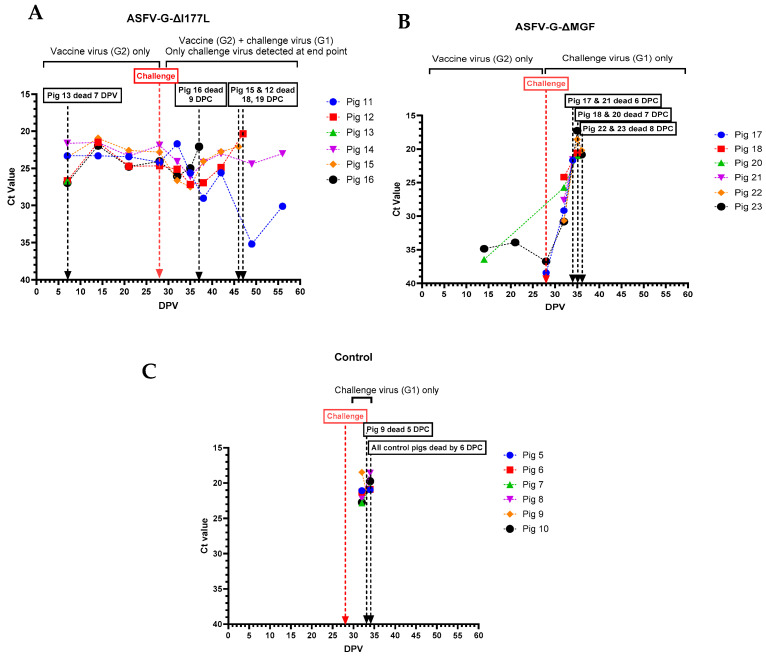
Viremia was detected in individual pigs following inoculation with ASFV-G-ΔI177L (**A**), ASFV-G-ΔMGF (**B**), or sterile PBS (**C**) followed by the rASFV I/II challenge. The samples with detectable levels of ASFV viral genome were further tested to differentiate the genotype II (G2) vaccinated strains vs. the recombinant challenge virus strain (G1). DPV = days post-vaccination.

All pigs that received the ASFV-G-ΔI177L developed viremia and remained viremic until they were challenged with rASFVI/II (Figure 6A). Pigs #12, 15, and 16 that succumbed to ASF after the challenge developed high viremia. Pig #14, which had fever, lethargy, and swollen hock joints, also had a high level of viremia until the end of the study. In contrast, pig #11 had low levels of viremia by the end of the study. Only two (#20 and 23) out of six pigs that received ASFV-G-ΔMGF developed a detectable level of viremia before the challenge (Figure 6B). After the challenge, all six pigs developed viremia within 4 days, and the viremia continued to increase until they were found dead/humanely euthanized. All pigs in the control group developed high levels of viremia and succumbed to ASF by 6 dpc (Figure 6C).

In order to determine if the viremia developed after the challenge was caused by the vaccine strains (p72 genotype II) or rASFV I/II (p72 genotype I), all ASFV RT-PCR positive samples were subjected to Phan G1 and G2 qRT-PCR. Phan G1 and G2 qRT-PCR assays were specific but slightly less sensitive than the Tignon assay (Supplementary Materials Table S2). Whole blood samples from pigs that received ASFV-G-ΔI177L tested positive by both Phan G1 and G2 RT-PCR assays, except those collected on the last day of the study. Whole blood from pigs #11 and 14 collected before euthanasia tested positive only in the Phan G1 assay.

**Figure 7:**
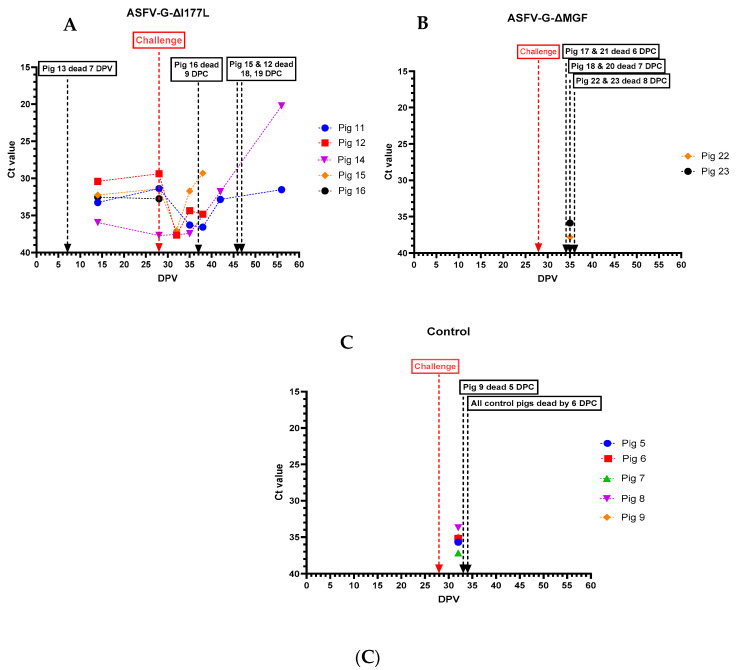
Detection of ASFV genomic material in nasal swabs collected from individual pigs following inoculation with ASFV-G-ΔI177L (**A**), ASFV-G-ΔMGF (**B**), or sterile PBS (**C**) followed by the rASFV I/II challenge. DPV = days post-vaccination.

ASFV genomic material was detected in nasal swabs collected from pigs that received ASFV-G-ΔI177L, starting at 15 dpv. The highest amount of ASFV genome was detected in the nasal swab collected from pig #14 on the day it was euthanized (Figure 7A). No ASFV genome was detected in nasal swabs collected from pigs that received the AVAC ASFV-G-ΔMGF strain until they were challenged with rASFV I/II (Figure 7B). Similarly, the ASFV genome was positive (low levels) in nasal swabs collected from control pigs only after they were challenged with rASFV I/II (Figure 7C).

### 3.3. Anti-ASFV Antibodies

**Figure 8:**
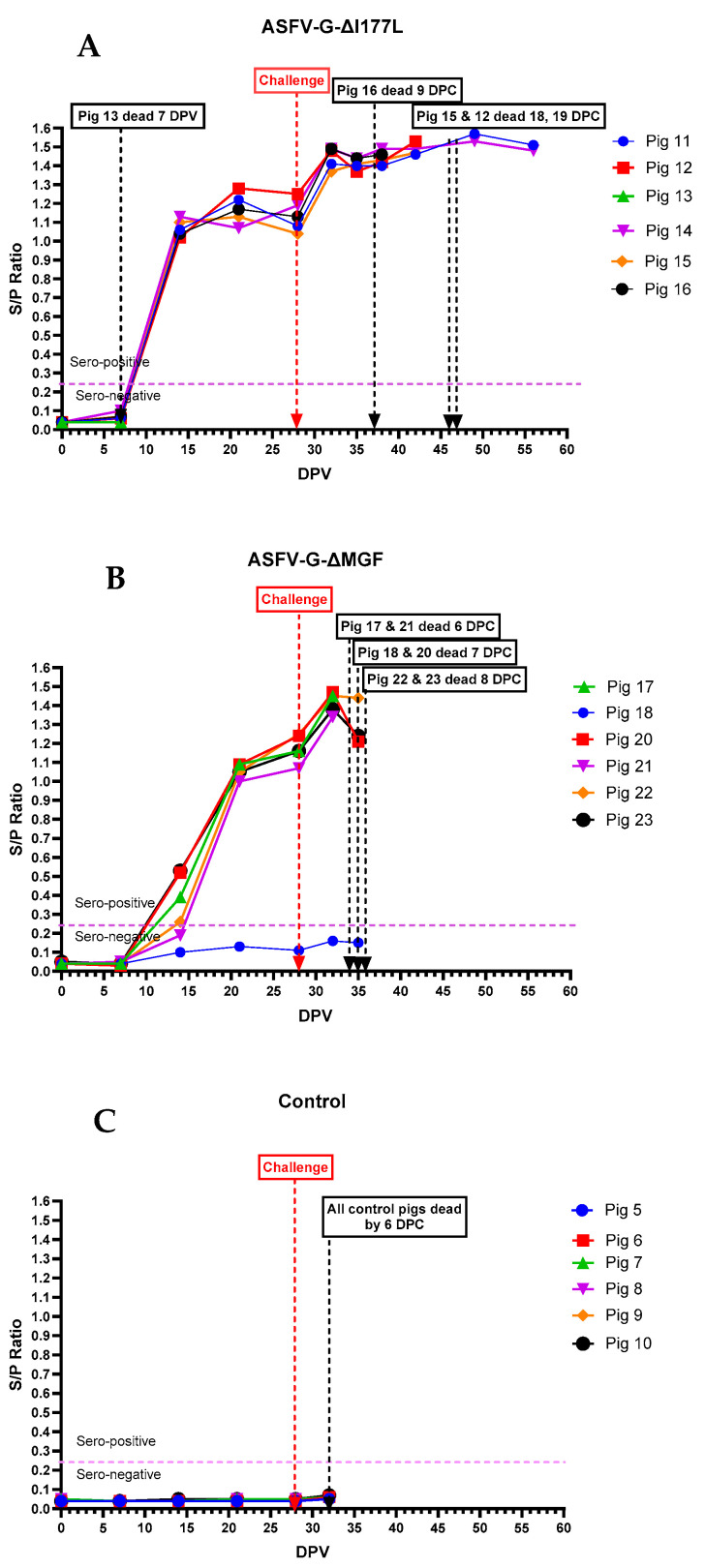
Serum anti-ASFV antibody levels in individual pigs following inoculation with ASFV-G-ΔI177L (**A**), ASFV-G-ΔMGF (**B**), or sterile PBS (**C**) followed by the rASFV I/II challenge.

All pigs that received ASFV-G-ΔI177L, except pig #13, which died at 7 dpv, developed anti-ASFV antibodies by 14 dpv (Figure 8A). Anti-ASFV antibodies continued to increase in those pigs and plateaued around 20 dpv. Following the rASFVI/II challenge on 29 dpv, the antibodies increased again and remained high until the end of the experiment. In contrast, pigs that received the AVAC ASFV-G-ΔMGF vaccine strain developed anti-ASFV antibodies relatively slowly compared to those that received ASFV-G-ΔI177L (Figure 8B). Interestingly, pig #18 did not develop detectable levels of anti-ASFV antibodies even after the challenge. As expected, none of the pigs in the control group developed detectable levels of anti-ASFV antibodies until they succumbed to the rASFV I/II challenge.

### 3.4. Postmortem Findings

**Figure 9:**
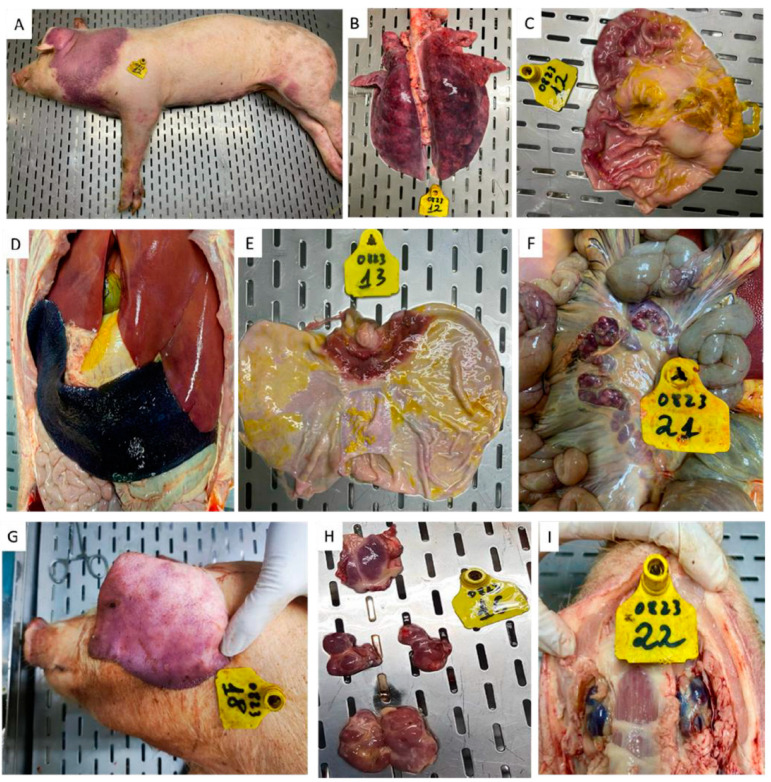
Gross pathological lesions observed in pigs that received ASFV-G-ΔI177L (**A**–**E**), ASFV-G-ΔMGF (**F**–**I**), and died/were euthanized after the rASFV I/II challenge. (**A**) Subcutaneous hemorrhage and hematoma in the left pinna, (**B**) consolidated lungs, (**C**) congestion in the gastric mucosa, (**D**) splenomegaly, (**E**) ulceration at the gastroesophageal junction, (**F**) hemorrhagic mesenteric lymph nodes, (**G**) hemorrhages in the ear, (**H**) hemorrhages in the tonsil, submandibular lymph nodes, and superficial inguinal lymph nodes, and (**I**) hemorrhagic submandibular lymph nodes.

Pig #13 was the first to die in this study at 7 dpv. Other than the mucosal ulceration at the gastroesophageal junction (Figure 9E) and hemorrhagic submandibular lymph nodes, no other gross lesions were observed in this pig. Pigs #12, 15, and 16, which died after the rASFV I/II challenge, had subcutaneous hemorrhages in the skin (Figure 9A) and lymphoid tissues, splenomegaly (Figure 9D), consolidated lung lobes (Figure 9B), and congested gastric mucosa (Figure 9C).

**Figure 10:**
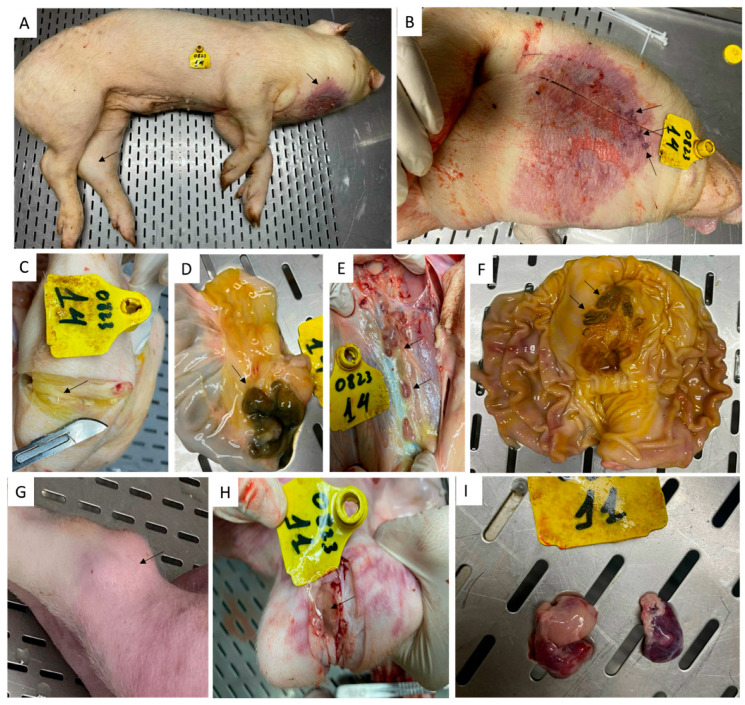
Gross pathological lesions observed in pigs #14 (**A**–**F**) and 11 (**G**–**I**) that received ASFV-G-ΔI177L and survived the rASFV I/II challenge. (**A**) Swollen left hock joint (arrow) and dermatitis under the neck, (**B**) Dermatitis with necrotic and denuded skin, (**C**) Increased synovial fluid in the left hock joint, (**D**) Necrotic lymphoid tissues of the ilea-cecal valve, (**E**) Hemorrhagic lymph nodes, (**F**) Gastric ulcers (arrows), (**G**) Swollen hock joint, (**H**) Increased synovial fluid in the hock joint, (**I**) hemorrhagic submandibular lymph nodes.

Among the two pigs that survived the rASFV I/II challenge, pig #14 had the most gross pathological lesions (Figure 10). Under the neck, it had severe dermatitis with denuded and necrotic skin lesions (Figure 10A,B). During the postmortem, gastric ulcers (Figure 10F), hemorrhagic lymph nodes (Figure 10E), necrotic ileocecal lymph nodes (Figure 10D), and increased synovial fluid in the swollen hock joints (Figure 10C) were observed. In contrast, pig #11 only had a swollen hock joint with increased synovial fluid (Figure 10G,H) and hemorrhagic submandibular lymph nodes (Figure 10I). A selected number of tissues from pigs #11 and 14 was tested by RT- PCR, and pig #14 had the highest amount of viral genomic material in the tissues compared to pig #11 (Table 1). When using Phan G1 and G2 assays, both vaccine (G2) and challenge (G1) viruses were detected in lymphoid tissues collected from the two pigs.

In controls (pigs #5–10) and pigs that received ASFV-G-ΔMGF (pigs #17–23), hemorrhages on the skin (Figure 9G) and in internal lymphoid organs (Figure 9F,H,I) were observed.

## 4. Discussion

To date, there is no globally approved vaccine available for ASF; however, live-attenuated ASFV strains are considered the most promising vaccine candidates available thus far. Several live attenuated ASFV strains, including HLJ/18-7GD [25], ASFV-G-ΔI177L [22], and ASFV-G-ΔMGF [20], have been shown to induce complete protection against homologous high virulent ASFV challenge under experimental conditions. Of those, ASFV-G-ΔI177L and ASFV-G-ΔMGF strains have been licensed as NAVET-ASFVAC^®^ and AVAC ASF LIVE^®^ in Vietnam, respectively. In this study, we showed that ASFV-G-ΔI177L and ASFV-G-ΔMGF strains induced strong immune response (based on antibody response) as demonstrated previously [20,22], but were not able to effectively protect the pigs from the emerging rASFV I/II in Vietnam.

In this study, we used 18 specific pathogen-free weaned piglets. They were inoculated with ASFV-G-ΔI177L (6 pigs) or DMAC passaged ASFV-G-ΔMGF (6 pigs). Six pigs were used as controls, and they received sterile PBS intramuscularly. Out of the 6 pigs that received DMAC passaged ASFV-G-ΔMGF, none showed ASF-related clinical signs, and 2 developed low levels of viremia. There was no virus shedding observed in nasal secretions. The findings were different from those reported by O’Donnell et al. [19] and Deutschmann et al. [26] with the original ASFV-G-ΔMGF strain. In both studies, all pigs that received the ASFV-G-ΔMGF (IM at 10^4^ HAD_50_/pig) developed viremia, and some shed the virus in secretions and excretions. The difference observed could be due to further attenuation of the ASFV-G-ΔMGF strain because of multiple passages in the DMAC cell line. All pigs that received the ASFV-G-ΔI177L strain developed viremia and shed virus in their nasal secretions. Four pigs, including pig #13, which died at 7 dpv, developed a fever. Pig #13 had the highest level of viremia, but there were no ASF-related lesions other than gastric erosions observed at the postmortem. Therefore, it was not clear if the pig died of ASF or due to another cause.

When challenged, the control pigs developed severe clinical signs and succumbed to ASF by 6 dpc, confirming the highly pathogenic nature of the rASFV I/II. Similarly, pigs vaccinated with the AVAC ASFV-G-ΔMGF strain when challenged died within 8 dpc despite high antibody levels observed in five out of six pigs. In contrast, in pigs that received the ASFV-G-ΔI177L strain, the development of ASF clinical signs was delayed. Only three pigs (#12, 15, and 16) developed acute clinical signs and succumbed to ASF at 19, 18, and 9 dpc, respectively. The two remaining pigs (#11 and 14) survived but started to show signs of chronic ASF infection as they reached the end of the study. The lymphoid tissues from pigs #11 and 14 contained ASFV genomes from the vaccine and challenge viruses. Chronic ASF was characterized by necrotic dermatitis and swollen joints and has been reported exclusively in pigs infected with low-virulent ASFV genotype I viruses. Therefore, one can speculate that these lesions could be caused by the proteins encoded by ASFV genotype I genes in the rASFV I/II strain.

The findings from this study are in line with those reported by Zhao et al. [13]. They used a p72 genotype II virus-based vaccine, HLJ/18-7GD, which lacks seven ASFV genes, to vaccinate the pigs and challenged them with one of the three rASFV I/II viruses isolated from China. The vaccine was able to induce a substantial antibody response and protect the pigs from ASF when challenged with a highly virulent p72 genotype II ASFV HLJ/18 strain. However, the vaccine was not able to protect the vaccinated pigs against the recombinant virus JS/LG/21 challenge. All vaccinated pigs developed fever and succumbed to ASF within 10 days post-challenge. In another study, the HLJ/18-7GD vaccine failed to provide solid cross-protection against genotype I low virulent isolate SD/DY-I/21, detected in China in 2021 [25]. Overall, these studies show that the immune response induced by attenuated p72 genotype II ASFV strains is able to protect pigs against virulent p72 genotype II strain challenges, but not when pigs are challenged with genotype I or ASFV genotype I and II recombinant viruses. The exact mechanism for the lack of cross-protection is not clear; however, the ASFV proteins with genotype I origin appear to be responsible.

To our knowledge, this is the first study that directly compared ASFV-G-ΔI177L and ASFV-G-ΔMGF vaccine strains in an in vivo experiment. Both strains were inoculated to pigs at the same dose (10^4^ HAD_50_ per pig) for direct comparison. The AVAC ASF LIVE^®^ single dose contains ~ 10^4^ HAD_50_ ASFV-G-ΔMGF; however, the NAVET-ASFVAC^®^ contains only ~10^3^ HAD_50_ ASFV-G-ΔI177L. Therefore, the viremia, clinical signs, and better immune response and enhanced protection observed in pigs that received ASFV-G-ΔI177L could be due to the 10 times the recommended dose of ASFV-G-ΔI177L used in this experiment. Compared to the AVAC ASFV-G-ΔMGF vaccine strain, The NAVET-ASFVAC^®^, when used as recommended, might not be able to delay the clinical signs and induce chronic ASF in some of the vaccinated pigs as observed in this study.

ASF-attenuated vaccine strains mimic the natural infection and therefore induce a strong and long-lasting immune response. They need to replicate enough to induce protective immunity but not too much to cause a cytokine storm that leads to pathological lesions eventually killing the pig. Therefore, increasing the ASFV-G-ΔMGF dose or boosting the pigs might help to increase resistance to the rASFV I/II challenge. However, this might not prevent the development of chronic ASF in the vaccinated animals, as observed in pigs that received the ASFV-G-ΔI177L strain.

Due to the highly virulent nature and resistance to the licensed vaccines in Vietnam, the emerging rASFV I/II strain could soon replace the genotype II pandemic ASF viruses circulating in the region, derailing the ongoing efforts to control the ASF pandemic. Hence, there is an urgent need to develop a novel vaccine based on rASFV I/II for effective control of ASF in Asia. Targeted deletion of I177L or MGF genes from the rASFV I/II might result in an effective vaccine strain; however, due to the presence of genotype I genetic elements, such a vaccine could give rise to complications such as arthritis and skin lesions, as observed when p72 genotype I vaccines were used to control the ASF genotype I outbreak in Europe. Little is known about the pathogenesis of chronic ASF. The ability to induce chronic ASF in pigs in a short time by ASFV-G-ΔI177L vaccination followed by rASF I/II challenge might provide a model for those studies.

In conclusion, the findings from this study show that both vaccine strains licensed in Vietnam are unlikely to effectively protect pigs from the emerging highly virulent rASFV I/II strain. This complicates the ongoing efforts to control ASF in Asia and globally and emphasizes the urgent need for a novel vaccine that can protect pigs from the rASFV I/II strain.

## Figures and Tables

**Table 1:** Detection of ASFV genomic material in tissues collected from pigs #11 and 14 that survived the rASFV I/II challenge using the Median Diagnostics VDx ASFV qPCR kit, Phan G1, and Phan G2 RT-PCR assays.

Sample	Pig #11			Pig #14		
	VDx ASFV	Phan G1	Phan G2	VDx ASFV	Phan G1	Phan G2
Joint fluid	25.01	29.66	35.33	18.75	23.18	-
Tonsils	37.35	-	-	22.36	27.55	33.30
Thoracic lymph node	30.13	35.61	-	22.54	29.27	30.70
Submandibular lymph node	32.12	-	-	19.16	23.44	28.12
Mesenteric lymph node	32.49	-	-	25.67	30.40	-
Renal lymph node	26.30	30.56	-	24.05	30.08	30.50
Liver	35.76	-	-	27.15	31.94	-
Kidney	36.02	-	-	30.66	34.53	-
Spleen	30.48	34.95	-	27.39	32.66	-

## Data Availability

The data presented in this study are available on request from the corresponding author.

## Supplementary Materials

The following supporting information can be downloaded at: https://www.mdpi.com/article/10.3390/vaccines12101114/s1, Table S1: In-house developed scoring system used in the study; Table S2: The primer and probe sequences of the Phan G1 and G2 RT-PCR assays (A), and the performance of the two assays compared to published Tignon and G1 ACDP RT-PCR assays against a dilution series (in PBS) of ASFV Malata’78 (p72 genotype I) and ASFV Georgia 2007/1 (p72 genotype II) amplified in PPL cultures (B).
