# Supplementary material for: Genotype II Live-Attenuated ASFV Vaccine Strains Unable to Completely Protect Pigs against the Emerging Recombinant ASFV Genotype I/II Strain in Vietnam
Source: Vaccines (Basel). 2024 Sep 28;12(10):1114. doi: 10.3390/vaccines12101114 (PMC11511409; doi:10.3390/vaccines12101114)
Supplement: Supplementary file 1 [file vaccines-12-01114-s001.zip › vaccines-3188200-supplementary.pdf]

Supplementary Files – Genotype II Live-Attenuated ASFV Vaccine Strains Unable to Completely Protect Pigs against the Emerging Recombinant ASFV Genotype I/II Strain in Vietnam

Table S1. In-house developed scoring system used in the study.

Clinical signs	Severity level	Points
1: Temperature	<40.0	0
	40.0≤ to <40.5	1
	40.5≤41	2
	>41	3
2: Anorexia	Normal appetite	0
	Mild (reduced eating)	1
	Moderate (picking at feed)	2
	Severe (no interest in feed)	3
3: Recumbence	No abnormality	0
	Mild (stillness)	1
	Moderate (get up only when encouraged)	2
	Severe (remain recumbent when encouraged)	3
4: Skin Hemorrhage/ Cyanosis	No abnormality	0
	Mild	1
	Moderate	2
	Severe	3
5: Joint Swelling	No abnormality	0
	Mild (joint swelling)	1
	Moderate (Joint swelling + lameness)	2
	Severe (severe lameness, impaired walking)	3
6: Labored breathing and/or coughing	No abnormality	0
	Mild	1
	Moderate	2
	Severe	3
7: Ocular discharge	No abnormality	0
	Mild	1
	Moderate	2
	Severe	3
8: Digestive findings	No abnormality	0
	Mild (diarrhea<24 h)	1
	Moderate (diarrhea >24 h + vomiting)	2
	Severe (bloody diarrhea)	3

Table S2. The primer and probe sequences of the Phan G1 and G2 RT-PCR assays (A), and the performance of the two assays compared to published Tignon and G1 ACDP RT-PCR assays against a dilution series (in PBS) of ASFV Malata'78 (p72 genotype I) and ASFV Georgia 2007/1 (p72 genotype II) amplified in PPL cultures (B). NTC = No template control. Ext. Ctr. = Extraction control.

A

**Name**	**Sequence**
Forward Primer	5'-ATTGTTGGTGTGGGTCACCT-3'
Reverse Primer	5'-GGATTTGGTGAATGAATTTCCTGG-3'
Phan G1 Probe	5′- Q670/ACGCCAGTCGCGTTTTATACC/BHQ2-3′
Phan G2 Probe	5′- FAM/ACGTATCAGCGAAAAGCGAACG/ BHQ1-3′

B

**Virus**	**Dilution**	**Tignon**	**G1 ACDP**	**Phan G1**	**Phan G2**	**β-actin**
**ASFV Matla'78**	10^{0}	23.9	22.3	25.3	0	29.4
	10^{-1}	27.9	26.31	28.7	0	33.1
	10^{-2}	30.9	29.64	32.0	0	35.8
	0^{-3}	35. 0	33.08	35.4	0	0
	10^{-4}	35.9	35.25	37.3	0	0
	10^{-5}	38.1	0	0	0	0
	10^{-6}	0	0	0	0	0
**ASFV Georgia 2007/1**	10^{0}	23.9	0	0	23.6	33.8
	10^{-1}	26.6	0	0	26.2	30.2
	10^{-2}	33.4	0	0	32.7	37.5
	10^{-3}	35.9	0	0	35.3	0
	10^{-4}	37.7	0	0	0	0
	10^{-5}	0	0	0	0	0
	Ext. Ctrl.	0	0	0	0	0
	NTC	0	0	0	0	0
